# IL‐6 promotes metastasis of non‐small‐cell lung cancer by up‐regulating TIM‐4 via NF‐κB

**DOI:** 10.1111/cpr.12776

**Published:** 2020-02-05

**Authors:** Wen Liu, Hongxing Wang, Fuxiang Bai, Lu Ding, Yanyan Huang, Changchang Lu, Siyuan Chen, Chunyang Li, Xuetian Yue, Xiaohong Liang, Chunhong Ma, Liyun Xu, Lifen Gao

**Affiliations:** ^1^ Key Laboratory for Experimental Teratology of Ministry of Education and Department of Immunology Shandong Provincial Key Laboratory of Infection & Immunology School of Basic Medical Sciences Shandong University Jinan Shandong China; ^2^ Laboratory for Tissue Engineering and Regeneration School of Stomatology Shandong University Jinan Shandong China; ^3^ Cell and Molecular Biology Laboratory Zhoushan Hospital Zhoushan Zhejiang China; ^4^ Department of Anatomy and Histoembryology School of Basic Medical Sciences Shandong University Jinan Shandong China; ^5^ Department of Cell Biology School of Basic Medical Sciences Shandong University Jinan Shandong China

**Keywords:** IL‐6, metastasis, NF‐κB, non‐small‐cell lung cancer, TIM‐4

## Abstract

**Objectives:**

Interleukin‐6 (IL‐6) is critical for the development of non‐small‐cell lung cancer (NSCLC). Recently, we identified T‐cell immunoglobulin domain and mucin domain 4 (TIM‐4) as a new pro‐growth player in NSCLC progression. However, the role of TIM‐4 in IL‐6‐promoted NSCLC migration, invasion and epithelial‐to‐mesenchymal transition (EMT) remains unclear.

**Materials and Methods:**

Expressions of TIM‐4 and IL‐6 were both evaluated by immunohistochemical staining in NSCLC tissues. Real‐time quantitative PCR (qPCR), Western blot, flow cytometry and RT‐PCR were performed to detect TIM‐4 expression in NSCLC cells with IL‐6 stimulation. The roles of TIM‐4 in IL‐6 promoting migration and invasion of NSCLC were detected by transwell assay. EMT‐related markers were analysed by qPCR and Western blot in vitro, and metastasis was evaluated in BALB/c nude mice using lung cancer metastasis mouse model in vivo.

**Results:**

High IL‐6 expression was identified as an independent predictive factor for TIM‐4 expression in NSCLC tissues. NSCLC patients with TIM‐4 and IL‐6 double high expression showed the worst prognosis. IL‐6 promoted TIM‐4 expression in NSCLC cells depending on NF‐κB signal pathway. Both TIM‐4 and IL‐6 promoted migration, invasion and EMT of NSCLC cells. Interestingly, TIM‐4 knockdown reversed the role of IL‐6 in NSCLC and IL‐6 promoted metastasis of NSCLC by up‐regulating TIM‐4 *via* NF‐κB.

**Conclusions:**

TIM‐4 involves in IL‐6 promoted migration, invasion and EMT of NSCLC.

## 1 INTRODUCTION

Non‐small cell lung cancer (NSCLC) is one of the malignant tumours with the fastest increase in mortality despite the implementation of novel targeted therapies and chemotherapeutic regimes.^1^, ^2^ Metastasis is responsible for the poor prognosis of NSCLC.^3^, ^4^ Epithelial‐mesenchymal transition (EMT), as a key regulator of metastasis, involves a series of phenotypic and behavioural changes, which contributes to the transformation of early tumours into invasive malignant tumours.^5^, ^6^ EMT has been verified to be responsible for the development of NSCLC resistance to anti‐cancer agents.^7^, ^8^, ^9^ Therefore, early identification of novel prognostic molecular markers related to NSCLC metastasis and EMT is urgently required.

Our previous studies showed that T‐cell immunoglobulin domain and mucin domain 4 (TIM‐4), closely related to poor NSCLC prognosis, could promote the growth, proliferation and cell cycle progress of NSCLC cell lines through the interaction of its RGD motif.^10^ However, the role of TIM‐4 in NSCLC metastasis and EMT has not been reported. Moreover, TIM‐4 levels in NSCLC tissues are significantly elevated, while the expression levels in various lung cancer cell lines are relatively low,^10^ and the relevant mechanism is still unclear.

Interleukin‐6 (IL‐6) is an important cytokine for EMT and tumour metastasis of NSCLC.^11^, ^12^, ^13^ The increase of IL‐6 can cause the resistance to molecular targeted therapy in lung cancer,^14^, ^15^, ^16^ and circulating IL‐6 level may be a prognostic marker for patients with NSCLC.^17^ Moreover, a humanized anti‐IL‐6 antibody (ALD518) has been used to treat NSCLC.^18^ Our previous studies showed that IL‐6 could induce TIM‐4 expression in vitro.^10^ Here, we investigated the exact role of TIM‐4 in IL‐6‐induced NSCLC migration, invasion and EMT.

## 2 MATERIALS AND METHODS

### 2.1 Animals

Pathogen‐free BALB/c nude mice (5‐6 weeks old, male) bought from Vital River Laboratory Animal Technology Co., Ltd were randomly assigned to experimental groups and housed in home cages with an alternating 12 hours light and 12 hours dark cycle, water available, in the Animal Center of Shandong University under specific pathogen‐free conditions. All animal experiments were performed with the approval of the Committee on the Ethics and use of Animal Experiments of Shandong University.

### 2.2 Human samples

Non‐small‐cell lung cancer tissues samples were collected from 106 patients who had undergone surgery at Zhoushan Hospital (Zhejiang, China) from January 1 in 2011 to January 1 in 2014, without receiving preoperative chemo‐ or radiotherapy. The patients’ age, gender, smoking history, carcinoembryonic antigen (CEA), tumour size, histology type, pleural invasion, lymph node metastasis, stage, and grade were determined by a reviewer according to the medical records. Each tumour sample was classified according to the 8th edition tumour‐node‐metastasis (TNM) classification of lung cancer. Follow‐up duration was determined from the date of surgical treatment until the death. The study was approved by the ethics committee of Zhoushan Hospital.

### 2.3 In vivo lung cancer metastasis test

Migratory ability was determined with lung cancer metastasis test in BALB/c nude mice. Briefly, A549 cells were transfected with LV‐NC‐Luciferase (LV‐NC) or LV‐shTIM‐4‐Luciferase (LV‐shTIM‐4), respectively. Stable cell lines with TIM‐4 knockdown or control were obtained by a high concentration of puromycin selection (12 μg/mL) for 2 weeks, and subsequently, stable clones were maintained in culture with a low concentration of puromycin (1 μg/mL) for tumour bearing in BALB/c nude mice. Then, a tail‐vein injection model for metastasis was generated by injecting 2 × 10^6^ LV‐NC or LV‐shTIM‐4‐A549 cells in 200 μL normal saline per mouse into the tail veins of nude mice. Twenty four hours later, mice were randomly assigned to four groups: (a) LV‐NC group with PBS administration intraperitoneally (n = 4), (b) LV‐NC group with IL‐6 administration intraperitoneally (n = 3), (c) LV‐shTIM‐4 group with PBS administration intraperitoneally (n = 3) and (d) LV‐shTIM‐4 group with IL‐6 administration intraperitoneally (n = 3). And the dose of IL‐6 (dissolved in PBS) was 2.5 μg/kg once daily at 10 o'clock in the morning for the first 2 weeks, then 5 μg/kg once every 3 days throughout the next 4 weeks. After 6 weeks, mice were monitored using the IVIS Spectrum In Vivo Imaging System (PerkinElmer) of Advanced Medical Research Institute, Shandong University. Mice were intraperitoneally injected with 200 μL of D‐luciferin (150 mg/kg body weight, PerkinElmer) and anesthetized with isoflurane gas after 5 minutes. One minute later, mice entered the state of general anaesthesia, and then, they were transferred into the cage of the in vivo imaging system. The chest and abdomen of mice were completely exposed, and then, image calibration and visualization were performed using Living Image 4.2 software (PerkinElmer). At 7 weeks, mice were euthanized with an intraperitoneal injection of pentobarbital‐phenytoin solution in the lower right abdominal quadrant. Then lungs were isolated, weighed, fixed in 4% paraformaldehyde at least for 24 hours and embedded in paraffin. Pathological changes were evaluated in sections (5 μm) by H&E staining. Numbers of metastatic nodules per lung were counted by panoslice scanner system (Tykor).

### 2.4 Statistical analysis

GraphPad Prism 6.0 software was used for data analysis. Log‐rank test was utilized to compare the survival for different groups. A logistic regression model was applied to investigate the independent predictive factors responsible for TIM‐4 expression. Additionally, the correlation between TIM‐4 and IL‐6 was analysed by Spearman's rank correlation. *P*‐values of <0.05 were considered as statistically significant. Other quantitative values were presented as the mean ± SD. Represent data were from at least triple or duplicate independent experiments. The differences in mean values between two groups were analysed by the Student's *t* test. *P < .05, **P < .01, ***P < .001, ****P < .0001, ns: not significant.

Additional supporting information of MATERIALS AND METHODS could be found in the Appendix S1.

## 3 RESULTS

### 3.1 High expression of TIM‐4 was positively correlated with IL‐6 in NSCLC, which indicated poor prognosis

**Figure 1:**
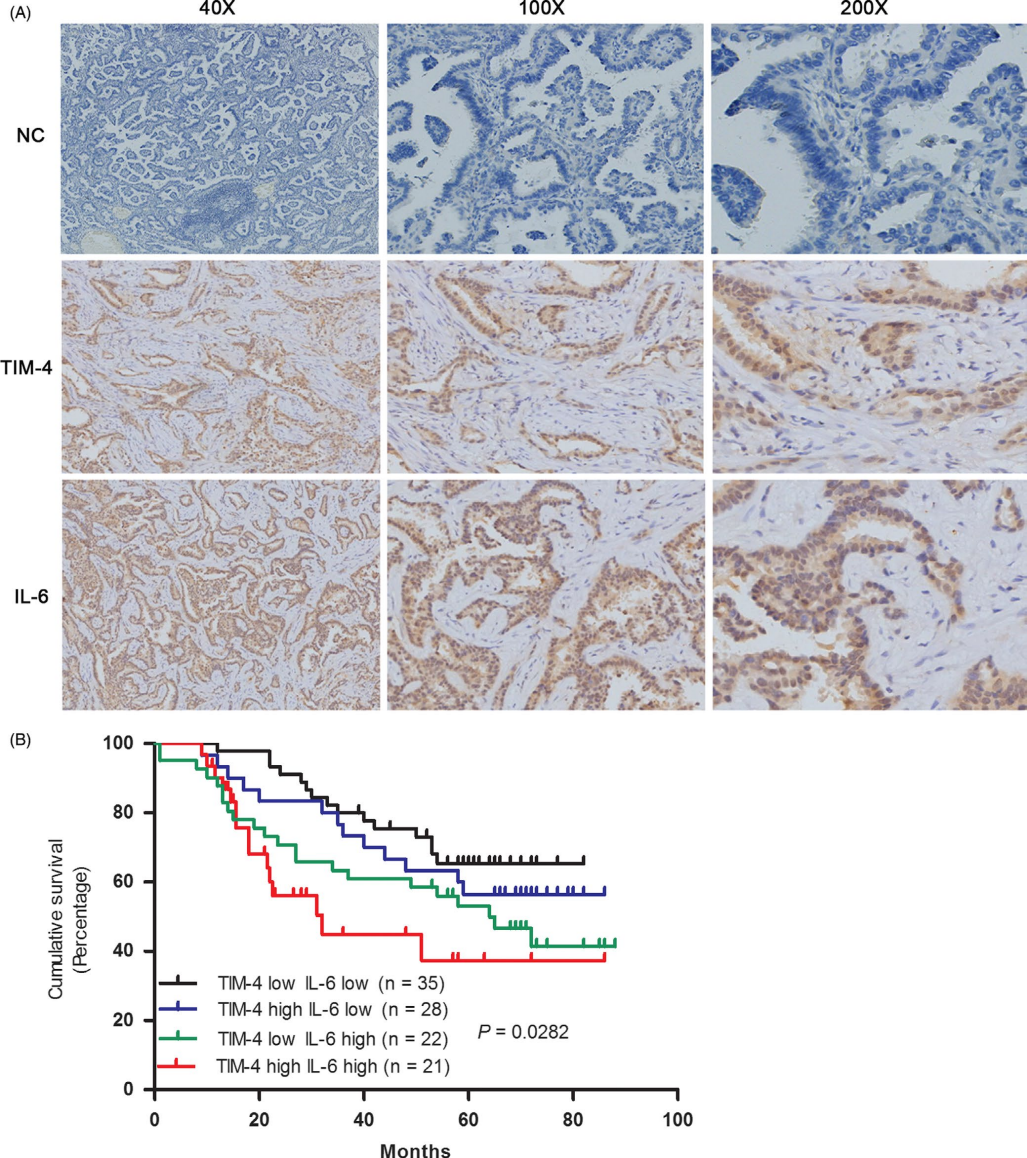
The prognostic significance of TIM‐4 and IL‐6 in tumour cells in patients with NSCLC. A, Representative positive immunohistochemical (IHC) staining for TIM‐4 and IL‐6 of NSCLC. B, Overall Survival (OS) by TIM‐4 and IL‐6

Previously, we found that TIM‐4 was highly expressed in cancer tissues of NSCLC.^10^ Here, we further investigated the predictive factors for TIM‐4 expression in clinical samples of NSCLC. Multivariate logistic regression analysis showed that tumour size (>3 cm) (OR, 2.574; 95% CI, 1.646‐4.026; *P* = .032), lymph node metastasis (OR, 2.011; 95% CI, 1.472‐3.744; *P* = .027) and high IL‐6 expression (OR, 2.951; 95% CI, 1.082‐6.085; *P* = .038) were independent predictive factors for TIM‐4 expression (Table 1). Subsequently, the correlation between TIM‐4 and IL‐6 were analysed separately, and it was shown that TIM‐4 expression was positively correlated with IL‐6 expression in NSCLC (Table 2, *r* = .489, *P* = .021). Representative stained fields on histopathological slides for TIM‐4 and IL‐6 were displayed in Figure 1A. Additionally, the combination of TIM‐4 and/or IL‐6 expressions was used to explore the relationships with prognosis. As shown in Figure 1B, patients with high TIM‐4 and IL‐6 expression showed the worst prognosis, while patients with TIM‐4 and IL‐6 double low appeared the best prognosis (*P* = .0282). These results suggested that TIM‐4 might be involved in the process of IL‐6 promoting the development of NSCLC.

**Table 1:** Multivariate logistic regression model for TIM‐4 expression in patients with non‐small cell lung cancer (N = 106)

Variables	Multivariate analysis		
	OR	95% CI	*P*
Age (>60 vs ≤60 y)	1.147	0.754‐1.722	.512
Gender (Male vs Female)	0.872	0.594‐1.340	.572
Smoking (Smoker vs Never‐smoker)	0.981	0.646‐1.498	.965
CEA(>5 ng/mL vs ≤5 ng/mL)	1.544	0.989‐2.342	.087
Tumour size (>3 cm vs ≤3 cm)	2.574	1.646‐4.026	**.032**
Histology type (Sq vs Ad)	1.324	0.920‐2.112	.064
PI (present vs absent)	1.462	0.931‐2.221	.426
LN (present vs absent)	2.011	1.472‐3.744	**.027**
Stage (I b‐ IV vs I a)	1.831	1.36 1‐2.678	.066
Grade (G2‐G4 vs G1)	1.482	0.986‐2.223	.078
IL‐6 expression (High vs Low)	2.951	1.082‐6.085	**.038**

Abbreviation: CEA, carcinoembryonic antigen; CI, confidence interval; HR, hazards ratio; LN, lymph node metastasis; PI, pleural invasion.

**Table 2:** Immunohistochemical scores of TIM‐4 and IL‐6 expression in NSCLC

TIM‐4 score	IL‐6 score			
	0	1	2	3
0	4	10	8	10
1	14	7	2	1
2	17	11	3	8
3	0	0	10	1

Note

*r* = .489, *P* = .021.

### 3.2 IL‐6 promoted TIM‐4 expression in NSCLC cell lines *via* NF‐κB pathway

**Figure 2:**
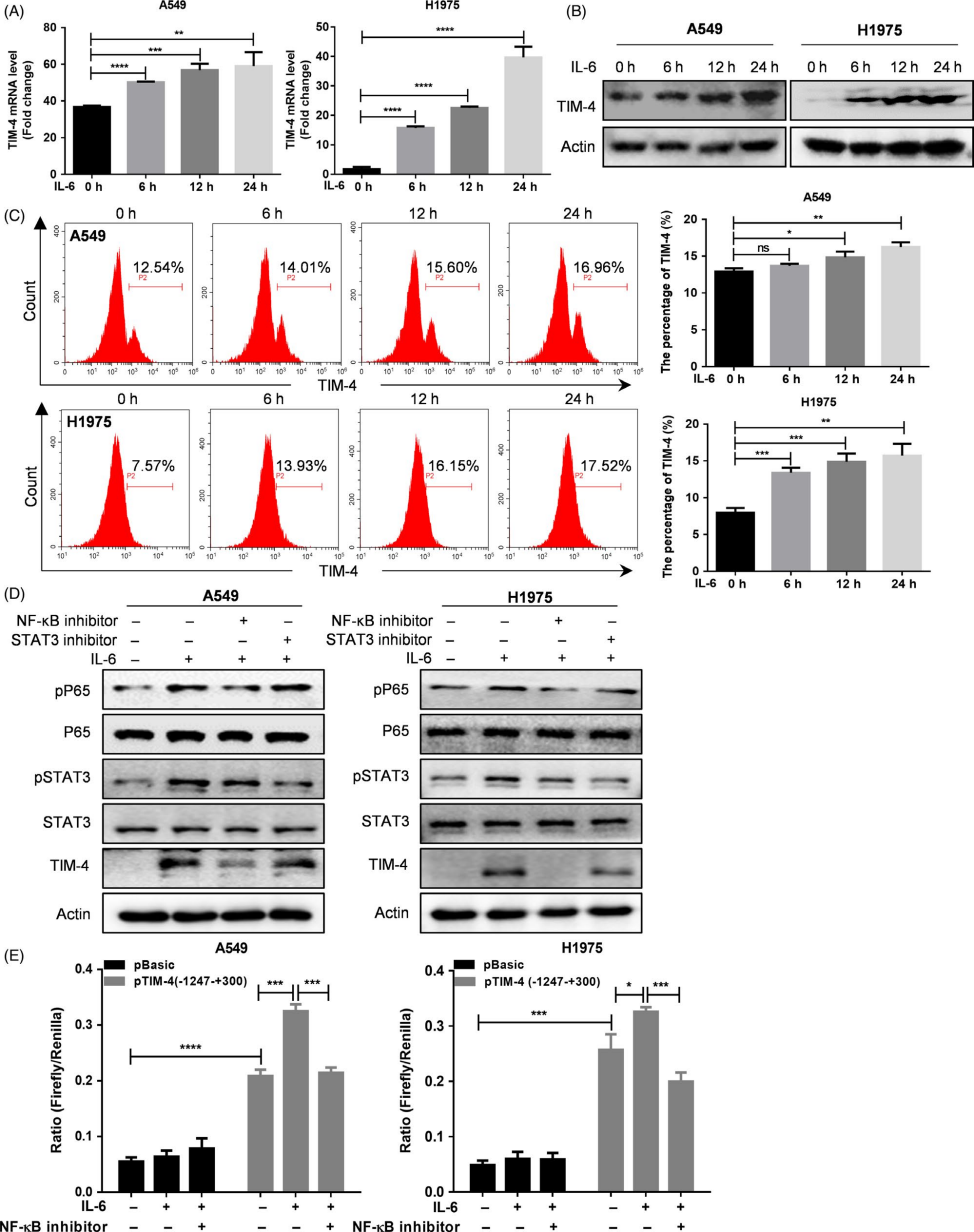
IL‐6 promoted TIM‐4 expression *via* NF‐κB pathway. IL‐6 was used to stimulate A549 and H1975 cells. TIM‐4 mRNA and protein levels were detected by qPCR (A), Western blot (B) and flow cytometry (C), respectively. D, NF‐κB or STAT3 inhibitor was used to incubate with IL‐6 stimulated A549 or H1975 cells, and phosphorylation of P65 or STAT3 and TIM‐4 protein expression were detected by Western blot. E, In A549 and H1975 cells, the TIM‐4 promoter activity was measured using a dual fluorescent reporter assay after stimulation with IL‐6, and IL‐6 plus NF‐κB inhibitor, respectively. The box plots in A, C and E showed median ± SD of three independent experiments. ns: no significance, *P < .05, **P < .01, ***P < .001, ****P < .0001, by 2‐tailed Student's t test

To verify that IL‐6 abundant in tumour microenvironment can induce high expression of TIM‐4, lung cancer cell lines (A549 and H1975) were treated with 50 ng/mL IL‐6 for the indicated time points (0, 6, 12 and 24 hours), and TIM‐4 expression was detected by qPCR, Western blot or flow cytometry, respectively. The results showed that IL‐6 could increase TIM‐4 expression at mRNA and protein levels in both A549 and H1975 cells in a time‐dependent manner (Figure 2A‐C). Furthermore, A549 and H1975 cells were stimulated by IL‐6 with different concentrations (0, 10, 50 and 100 ng/mL) for 24 hours, and RT‐PCR data demonstrated that both 10 and 50 ng/mL IL‐6 could increase the expression of TIM‐4 at mRNA level (Figure S1A). Subsequently, 50 ng/mL IL‐6 was used to stimulate lung cancer cells for 24 hours. Above all, the results showed that TIM‐4 expression in lung cancer cell lines was up‐regulated after IL‐6 stimulation.

It was reported that TIM‐4 inhibited cytokine production *via* NF‐κB signalling pathway^19^ and had no effect on STAT3 phosphorylation,^20^ while IL‐6 could increase the activation of NF‐κB^16^ and STAT3 signalling pathway.^21^ We then tested the changes of these signal molecules in IL‐6‐induced up‐regulation of TIM‐4 in lung cancer cells with NF‐κB inhibitor or STAT3 inhibitor, respectively. The results revealed that IL‐6 could increase the phosphorylation of p65 and TIM‐4 expression in A549 and H1975 cells, and the effects of IL‐6‐induced up‐regulation of TIM‐4 were decreased in NF‐κB inhibitor treatment group; however, IL‐6‐induced expression of TIM‐4 was slightly decreased in STAT3 inhibitor treatment group (Figure 2D). Taken together, these data suggested that NF‐κB might mediate IL‐6‐induced up‐regulation of TIM‐4 in NSCLC cells.

To verify whether IL‐6 promotes TIM‐4 promoter activity through transcription factor NF‐κB, we successfully constructed TIM‐4 promoter (−1247 to +300 bp) reporter plasmid (pGL3‐Basic‐hTIM‐4‐full fragment). Functional analysis of dual‐luciferase assay system both in A549 and H1975 cells showed that IL‐6 could enhance TIM‐4 promoter activity (Figure 2E). Then, we predicted and analysed the transcriptional factors associated with NF‐κB components and binding sites in TIM‐4 promoter (−1247 to +300 bp) by PROMO software and JASPAR software (Figure S1B). In accordance with the above prediction results, the effect of IL‐6 on promoting TIM‐4 promoter activity was attenuated after the addition of a specific inhibitor of NF‐κB (Figure 2E).

### 3.3 TIM‐4 overexpression promoted metastasis of NSCLC cells

**Figure 3:**
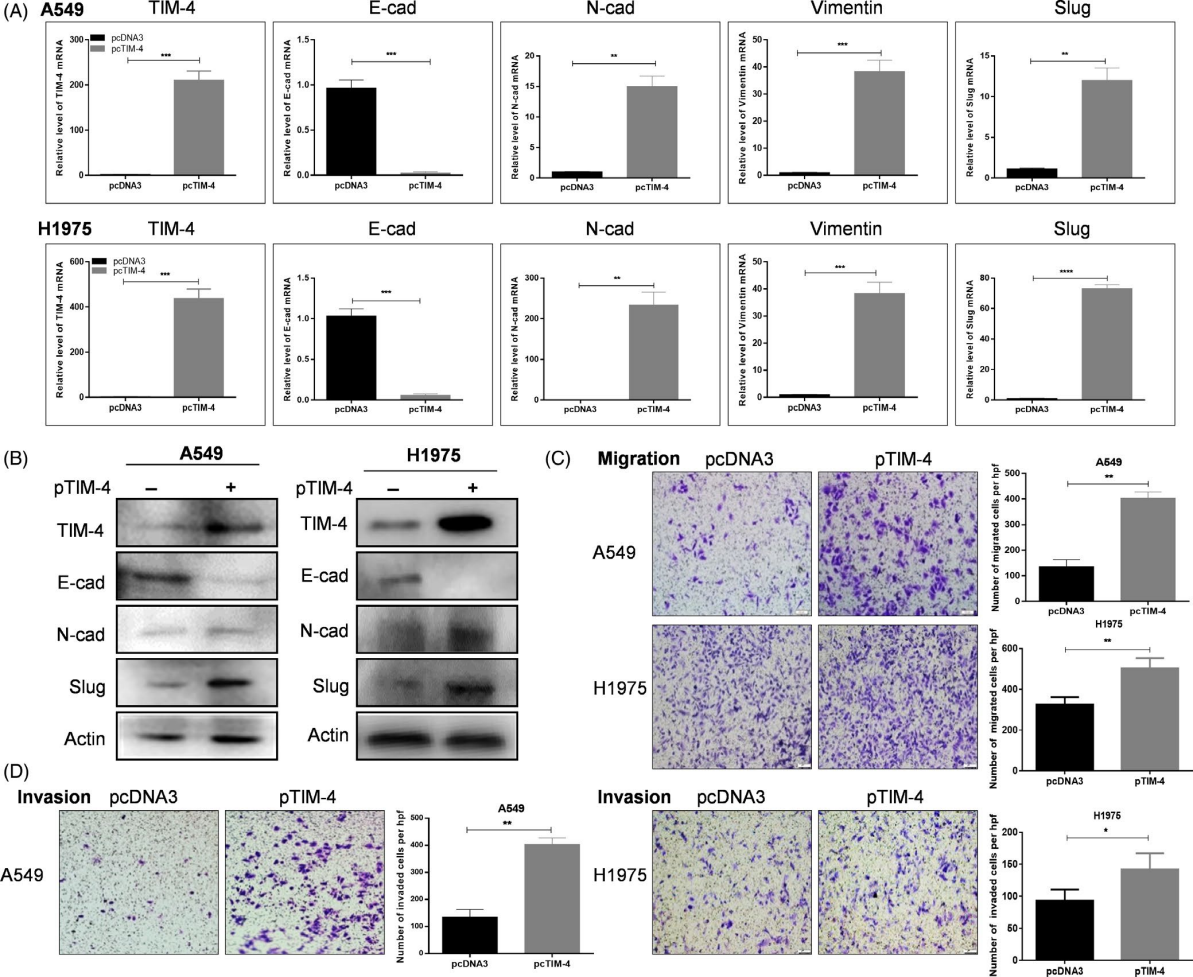
TIM‐4 overexpression promoted metastasis of lung cancer cells. EMT‐related genes E‐Cad, N‐Cad, vimentin and slug were assayed in TIM‐4 overexpressed A549 and H1975 cells by qPCR (A) and Western blot (B), migration (C) and invasion (D) by transwell assay. The migrated and invasive cells were photographed (Bar, 100 μm). Representative pictures were shown. Data in A, C and D were shown as median ± SD of three independent experiments. *P < .05, **P < .01, ***P < .001, ****P < .0001, by 2‐tailed Student's *t* test

Interestingly, we found that cell morphology of A549 and H1975 cells overexpressed with pcDNA3‐hTIM‐4‐HA (pTIM‐4) were more spindle‐like shape or fibroblast‐like than control cells (Figure S2A,B). The changes of cell morphology indicated that up‐regulated TIM‐4 expression might be associated with metastatic property of NSCLC cells. Many factors are involved in tumour metastasis, among which EMT is one of the key factors. Therefore, we investigated whether TIM‐4 overexpression in lung cancer cells regulated expression of molecules related to EMT. Then, A549 and H1975 cells were transfected with pTIM‐4 or pcDNA3 for 48 hours, respectively, and the EMT‐related genes were detected by qPCR and Western blot. The results showed that overexpressed TIM‐4 down‐regulated the epithelial marker, E‐cadherin and up‐regulated the levels of the mesenchymal markers, N‐cadherin, vimentin and slug both in A549 and H1975 cells (Figure 3A,B). These data suggested that TIM‐4 overexpression indeed significantly promotes EMT process of lung cancer cells.

Additionally, in the EMT process, tumour cells tend to appear much stronger abilities in migration and invasion, so we tested the effects of TIM‐4 on the migratory and invasive capabilities of A549 and H1975 cells by transwell assay. The results showed that overexpressed TIM‐4 in both A549 and H1975 cells promoted migration (Figure 3C) and invasion (Figure 3D) of A549 and H1975 cells in vitro. We further verified the role of TIM‐4 in EMT‐related experiments using a normal human bronchial epithelial cell line HBE, and the results were consistent with those in A549 and H1975 cells (Figure S3A‐D). All these data demonstrated that TIM‐4 promoted migration, invasion and EMT of NSCLC cells.

### 3.4 TIM‐4 increased IL‐6 production

To detect whether TIM‐4 knockdown affects the expression of IL‐6, we detected the attenuated IL‐6 expression in transcriptional level by RT‐PCR after interfering TIM‐4 (LV‐shTIM‐4‐GFP) in A549 cells (Figure S4A). We then tested the changes of signal molecules NF‐κB and STAT3 in A549 cells overexpressed TIM‐4 with NF‐κB inhibitor or STAT3 inhibitor, respectively. The results revealed that TIM‐4 promoted the phosphorylation of STAT3 and secretion of IL‐6 in A549 cells, but not phosphorylation of P65 (Figure S4B). However, TIM‐4‐induced up‐regulation of IL‐6 in A549 cells was slightly decreased in STAT3 inhibitor treatment group, but no change was found in NF‐κB inhibitor group (Figure S4B). Above all, TIM‐4 could also increase IL‐6 production in lung cancer cells, which might form a positive feedback loop in IL‐6 involved lung cancers.

### 3.5 IL‐6 promoted metastasis of NSCLC by up‐regulating TIM‐4 *via* NF‐κB in vitro

**Figure 4:**
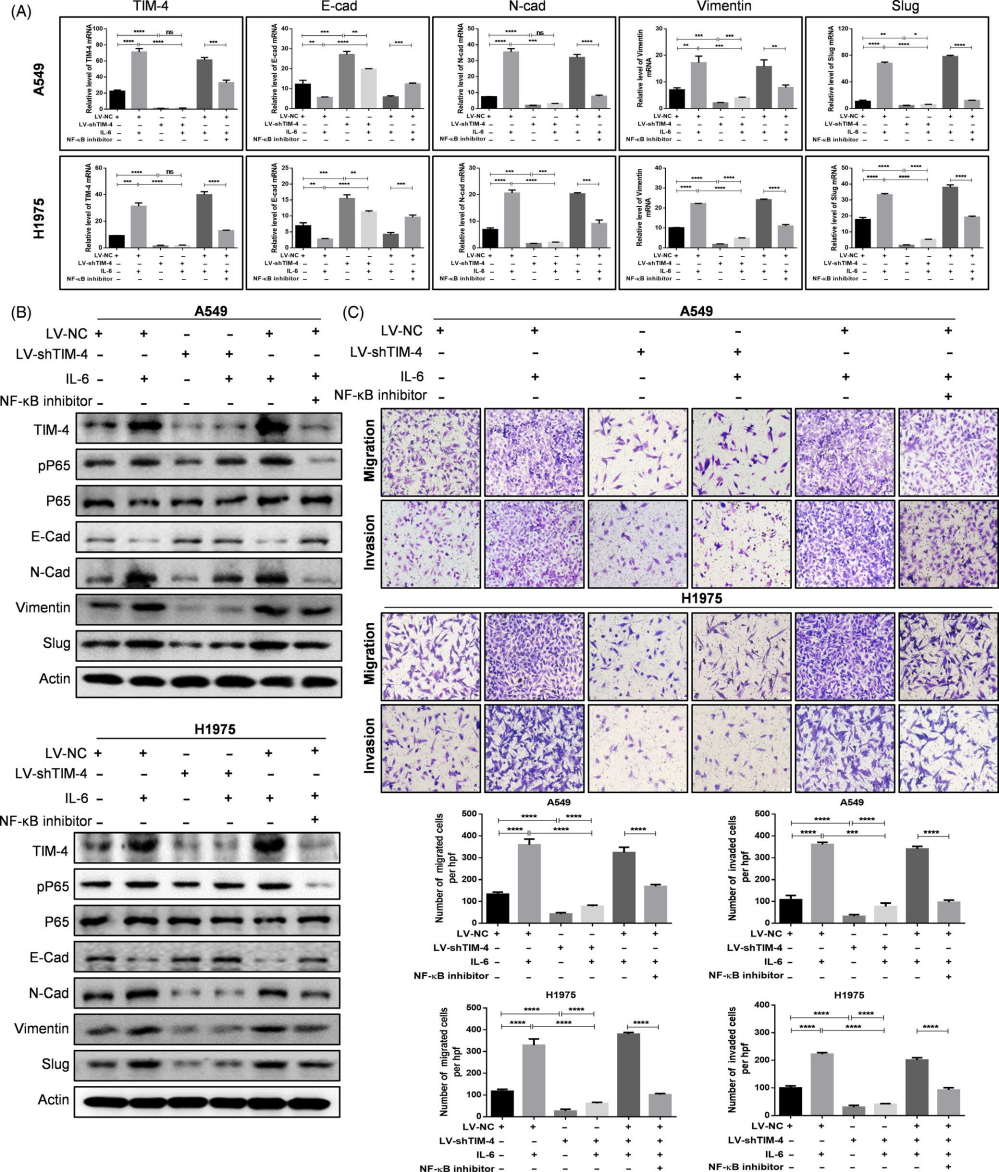
IL‐6 promoted metastasis of NSCLC by up‐regulating TIM‐4 *via* NF‐κB in vitro. EMT‐related markers and transwell assay were performed to evaluate the effects of TIM‐4 knockdown on IL‐6 promoting metastasis of A549 and H1975 cells. E‐Cad, N‐Cad, vimentin and slug were assayed in shTIM‐4‐A549 and H1975 cells with or without IL‐6 stimulation for 48 h and in LV‐NC plus IL‐6‐A549 and H1975 cells with or without NF‐κB inhibitor by qPCR (A) and Western blot (B), respectively. (C) Migration and invasion abilities were assayed in shTIM‐4‐A549 and H1975 cells with or without IL‐6 stimulation for 24 h and in LV‐NC plus IL‐6‐A549 and H1975 cells with or without NF‐κB inhibitor. The migrated and invasive cells were photographed. Representative pictures were shown (Bar, 50 μm). Data in A, B and C were shown as median ± SD of three independent experiments. ns: no significance, *P < .05, **P < .01, ***P < .001, ****P < .0001, by 2‐tailed Student's *t* test

Consistent with the report,^22^ we found that IL‐6 promoted migration and EMT of lung cancer cells (Figure S5A, B). To verify whether up‐regulated TIM‐4 was involved in IL‐6‐induced EMT of NSCLC in vitro, we knocked down TIM‐4 expression by shTIM‐4 lentivirus vector in both A549 and H1975 cells, and then, cells were stimulated with 50 ng/mL IL‐6 for 24 hours. Finally, we detected the effects of TIM‐4 on IL‐6 promoting lung cancer EMT capacity by qPCR and Western blot, migratory and invasive activities by transwell assay, respectively. The results showed that shTIM‐4 attenuated the mRNA and protein level of N‐cadherin, vimentin and slug, while increased the E‐cadherin expression. And IL‐6 up‐regulated the mRNA level of N‐cadherin, vimentin and slug, and down‐regulated E‐cadherin expression in both A549 and H1975 cells. However, the role of IL‐6 in promoting EMT of A549 and H1975 cells was weakened in shTIM‐4 group (Figure 4A,B). All these results indicated that TIM‐4 was involved in IL‐6‐induced EMT of NSCLC cells. To address the effects of TIM‐4 on IL‐6‐inducing the migration and invasion, we found that TIM‐4 knockdown significantly reversed IL‐6‐inducing migratory capacity and invasive activity by transwell assay (Figure 4C) both in A549 and H1975 cells. All these data revealed that TIM‐4 knockdown attenuated IL‐6‐promoting metastasis of NSCLC cells. As depicted in Figure 4, NF‐κB inhibitor significantly reduced the EMT ability, migratory and invasive capacities of lung cancer, as well as the elevated expression of TIM‐4 induced by IL‐6 stimulation, supporting the point that IL‐6 promoted metastasis of lung cancer by up‐regulating TIM‐4 *via* NF‐κB.

### 3.6 TIM‐4 knockdown inhibited IL‐6‐enhancing migration and invasion in lung cancer cells in vivo

**Figure 5:**
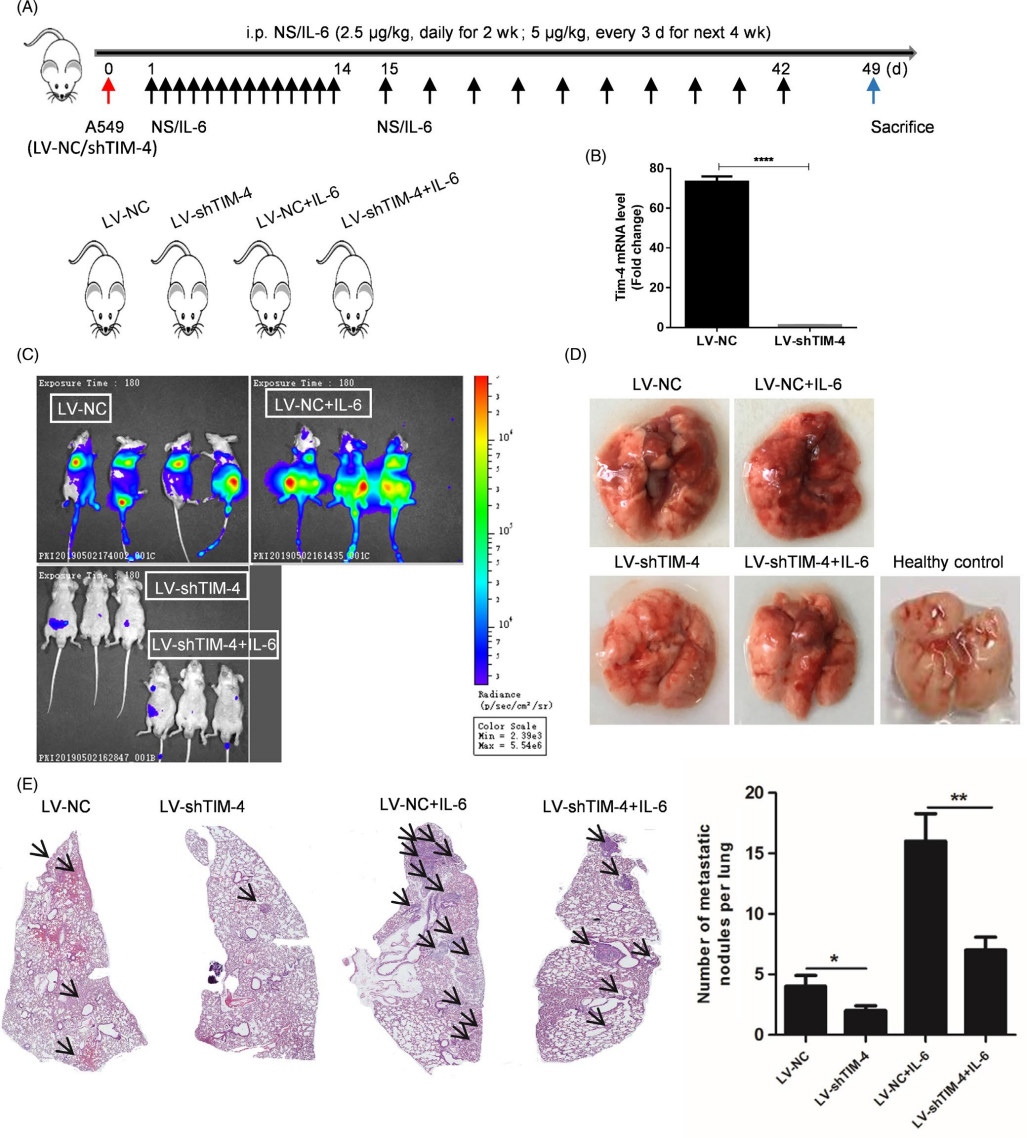
TIM‐4 knockdown attenuated IL‐6 promoting lung cancer metastasis in vivo. A, Experimental flow chart and design. B, Detection of TIM‐4 interference efficiency in lentivirus‐infected A549 cells by qPCR. C, Photographs of nude mice for in vivo imaging. D, Photographs of lung tissues. E, Scanning results of HE staining of lung tissues (left) and statistics of tumour nodules (right). Data in B and E were shown as median ± SD. *P < .05, **P < .01, ****P < .0001, by 2‐tailed Student's *t* test

**Figure 6:**
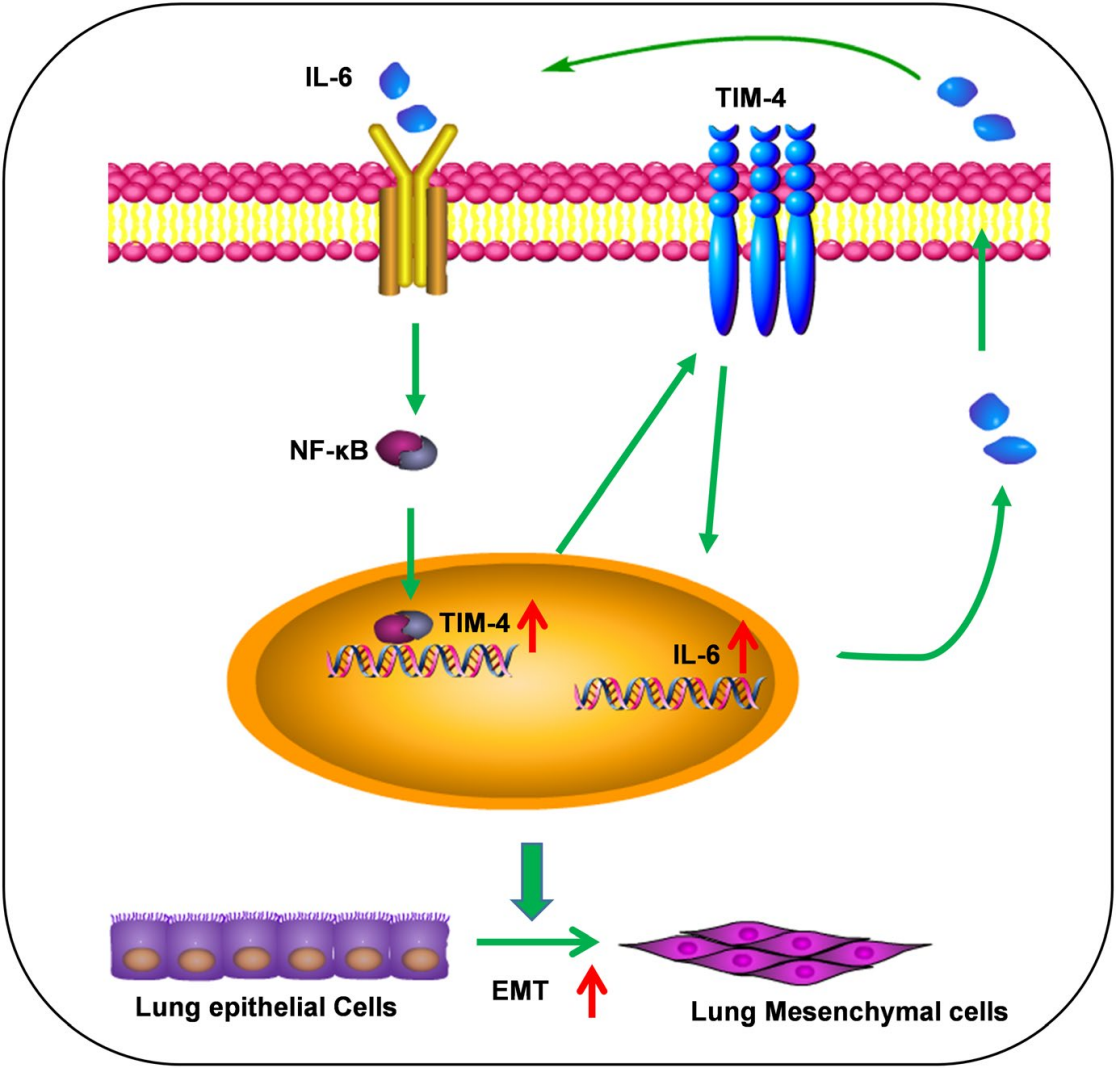
Working model for IL‐6 promoting metastasis of NSCLC by up‐regulating TIM‐4 *via* NF‐κB

The lung metastasis mice model was established to investigate whether knockdown of TIM‐4 could inhibit IL‐6‐enhancing metastasis of lung cancer cells in vivo. 2 × 10^6^ LV‐NC‐Luciferase‐A549 or LV‐shTIM‐4‐Luciferase‐A549 cells were injected into the tail vein of BALB/C nude mice with normal saline (NS) and IL‐6 administration, respectively. The experimental flow chart and grouping were shown in Figure 5A. Before injecting mice into the tail vein, we first verified the interference effect of TIM‐4 using qPCR (Figure 5B). 6 weeks later, we performed living imaging of mice in vivo. In NC group, the fluorescence was concentrated in the lungs. After IL‐6 stimulation, A549 cells carried the powerful metastatic ability from lungs to other organs and the intensity of fluorescence increased significantly. However, the fluorescence was almost vanished and not well transferred in TIM‐4 knockdown group in the presence or absence of IL‐6 administration (Figure 5C). Mice were sacrificed after 7 weeks of tumour bearing, and lung tissues were photographed. There were the most tumour nodules in IL‐6 administration group, and few tumour nodules were found in shTIM‐4 group (Figure 5D). In order to observe tumour nodules more intuitively, we performed HE staining with lung tissues. Metastatic nodules were counted in imaging scans, and the results showed that compared with NC group, mice in shTIM‐4 group showed decreased lung metastasis nodes, and mice in IL‐6 administration group showed a large number of lung metastasis nodules, while in shTIM‐4 plus IL‐6 group, lung metastasis nodules were increased slightly (Figure 5E). Though we did not inhibit NF‐κB signalling in vivo, we detected the elevated expression of pP65 and TIM‐4 in lung tissues from LV‐NC plus IL‐6 group by Western blot (Figure S6) simultaneously. These results indicated that TIM‐4 knockdown inhibited the role of IL‐6 in promoting metastasis of lung cancer cells in vivo. Above all, IL‐6 promoted migration, invasion and EMT of NSCLC by up‐regulating TIM‐4 *via* NF‐κB (Figure 6).

## 4 DISCUSSION

This is the first study to investigate the role of TIM‐4 in IL‐6‐inducing migration, invasion and EMT of NSCLC cells. We proved that high IL‐6 expression was an independent predictive factor for TIM‐4 expression in human NSCLC tissues. Importantly, our study showed that IL‐6 could up‐regulate TIM‐4 expression *via* the NF‐κB pathway in NSCLC cells. Moreover, TIM‐4 was identified to promote the abilities of migration, invasion and EMT of lung cancer cells. We further confirmed that TIM‐4 knockdown could inhibit IL‐6‐enhanced migration, invasion and EMT of lung cancer cells in vitro, as well as IL‐6‐enhanced lung cancer metastasis in vivo. This study provides a close link between TIM‐4 and IL‐6 in NSCLC, which suggests that TIM‐4 modulation might be a new mechanism of IL‐6‐promoting NSCLC progression.

TIM‐4 was initially found to be expressed only in antigen‐presenting cells. Our previous studies showed that LPS, IFN‐γ and ConA could enhance TIM‐4 expression in macrophages.^23^, ^24^ It was also reported that damage‐associated molecular patterns released from chemotherapy‐damaged tumour cells induced TIM‐4 expression on tumour‐associated macrophages and dendritic cells.^25^ Recent studies have shown that TIM‐4 is overexpressed in several tumours,^26^, ^27^ which is related with the worse prognosis of lung cancer and colorectal cancer.^10^, ^27^ Therefore, it was essential to explore the key factors regulating the abnormal expression of TIM‐4. Some cytokines, such as IL‐6, were accumulated in the tumour microenvironment and induced the expression of immune molecules, such as PD‐L1, which further aggravated the tumour development.^28^, ^29^, ^30^ Therefore, it was worth exploring whether TIM‐4 expression was regulated by IL‐6 in NSCLC cells. In clinical samples with NSCLC, we found that IL‐6 was an independent predictor of TIM‐4 expression, and patients with TIM‐4 and IL‐6 double high expression showed the worst prognosis. Importantly, we found that IL‐6 indeed increased TIM‐4 expression in lung cancer cell lines.

Next, we further examined the mechanism by which IL‐6 induced the expression of TIM‐4. As we know, NF‐κB is considered to be a central mediator of immune and inflammatory response, and IL‐6 could increase the activation of NF‐κB signalling pathway.^31^, ^32^, ^33^ In addition, Wang et al^32^ demonstrated that IL‐6‐induced intercellular adhesion molecule expression required NF‐κB activation, while our previous study showed that TIM‐4, acting as an adhesion molecule, bound to integrin to promote lung cancer growth.^10^ As expected, our study found that IL‐6 promoted TIM‐4 expression in NSCLC cell lines *via* NF‐κB pathway. In addition to NF‐κB, STAT3 is another key factor in IL‐6 signalling pathway,^34^, ^35^ and it has been documented that IL‐6 promoted lung cancer development and progression by activating STAT3.^36^, ^37^ Another study found that IL‐6 could induce immunosuppressive factor PD‐L1 expression on peripheral myeloid cells through STAT3‐dependent mechanism.^38^ However, we did not observe the significant change of IL‐6‐inducing elevated TIM‐4 expression in STAT3 inhibitor treatment group. It is interesting that TIM‐4 overexpression promoted IL‐6 production in lung cancer cells. Taken together, our data revealed the mechanism of up‐regulation of TIM‐4 expression in lung cancer cells, which would provide a new target for regulating TIM‐4 expression in tumour microenvironment.

Previously, we identified for the first time that TIM‐4 expression was augmented in NSCLC cancer tissues and was negatively correlated with the prognosis of patients.^10^ Also, it was shown that TIM‐4 overexpression promoted lung cancer cell growth and proliferation both in vitro and in vivo.^10^ However, it was not clarified the effect of TIM‐4 on migration, invasion and EMT of lung cancer cells. It is well known that EMT plays critical roles in the invasion and metastasis of cancer cells. Shan B et al^39^ reported that TIM‐3, another member of TIM family, promoted the metastasis of oesophageal squamous cell carcinoma by inducing EMT. Another study also showed that TIM‐3 facilitated osteosarcoma proliferation and metastasis through the NF‐κB pathway and EMT.^40^ Tan et al^27^ reported that TIM‐4 could drastically reduce E‐cadherin levels and promote the migration and invasion of colorectal cancer cells in vitro. Above results suggest a close association between TIM‐4 expression and metastasis. In this study, we found that lymph node metastasis was an independent predictive factor for TIM‐4 expression in lung cancer. As expected, we observed that TIM‐4 down‐regulated expression of EMT markers E‐cadherin, and up‐regulated expression of N‐cadherin, vimentin and slug in lung cancer cells. Moreover, we confirmed that TIM‐4 promoted migration, invasion and EMT of lung cancer cells in vitro, and TIM‐4 knockdown inhibited lung cancer metastasis in vivo. Our results further indicated that TIM‐4 was functioned as a tumour promoter in lung cancer. However, the molecular mechanism of TIM‐4 regulating EMT and then regulating metastasis requires to be further investigated.

Our published data reported that TIM‐4 could regulate cytokine production in macrophages.^24^ Whether TIM‐4 affects the expression of IL‐6 in lung cancer cells remains to be clarified. To this end, we tested IL‐6 levels in TIM‐4 knockdown A549 cells and found that IL‐6 expression was down‐regulated. Both NF‐κB and STAT3 pathways regulate IL‐6 production, which plays an important role in the development and prognosis of lung cancer. We demonstrated that TIM‐4 overexpression could lead to STAT3 activation in lung cancer cells, while TIM‐4 did not induce phosphorylation of NF‐κB. Therefore, we concluded that TIM‐4 increased IL‐6 production in lung cancer cells. However, the related mechanism requires further investigation. Our results were supported by those obtained by Zhang et al^41^ that Tim‐3 significantly up‐regulated the transcription and secretion of IL‐6 in liver cancer cells through NF‐κB/IL‐6/pSTAT3 axis.

Subsequently, we further confirmed that IL‐6 promoted migration of lung cancer cells, decreased E‐Cad and increased N‐Cad, which was consistent with IL‐6 promoting tumour metastasis. Based on the above results, we addressed whether up‐regulated TIM‐4 was involved in IL‐6‐inducing migration, invasion and EMT of NSCLC. We found that the effect of IL‐6 promoting EMT of lung cancer cells was weakened in TIM‐4 shRNA group, and TIM‐4 knockdown significantly reversed IL‐6‐induced migratory ability and invasive activity in vitro. Next, we also found that TIM‐4 knockdown inhibited IL‐6‐enhancing migration and invasion in an experimental lung metastasis mice model. Our data revealed that TIM‐4 may be an indispensable molecule involved in the progression of IL‐6‐overexpression tumours. Therefore, targeting TIM‐4 may offer a potential pharmacological target in IL‐6 overexpressed cancers.

In summary, the data presented in this study suggest that IL‐6 in tumour microenvironment enhance TIM‐4 expression in lung cancer cells, which in turn promotes metastasis and IL‐6 production of lung cancer cells. More importantly, up‐regulation of TIM‐4 is involved in IL‐6 promoted metastasis of NSCLC cells. The TIM‐4/IL‐6 positive feedback loop drives lung tumour metastasis. Our finding here highlights the potential role of TIM‐4 in enhancing metastasis of NSCLC and provides a new mechanism in IL‐6 promoting lung cancer progression.

## CONFLICT OF INTEREST

The authors declare no conflict interest.

## AUTHOR CONTRIBUTIONS

L. G. supervised all the subjects. L. G., L. X. and W. L. designed the experiments and wrote the manuscript. Y. H. and C. L. performed the experiments involving human data. W. L., H. W., F. B., L. D., S. C., C. L. and X. Y. performed the other experiments and analysed the data. X. L. and C. M. helped to design the experiments.

## Data Availability

The data that support the findings of this study are available within the article and its Appendix S1 or on reasonable request from the corresponding author.
