# Supplementary material for: IL‐6 promotes metastasis of non‐small‐cell lung cancer by up‐regulating TIM‐4 via NF‐κB
Source: Cell Prolif. 2020 Feb 5;53(3):e12776. doi: 10.1111/cpr.12776 (PMC7106962; doi:10.1111/cpr.12776)
Supplement: Supplementary file 1 [file CPR-53-e12776-s001.docx]

**Supplementary Information**

**IL-6 promotes metastasis of non-small-cell lung cancer by up-regulating TIM-4 *via* NF-κB**

Wen Liu^1^, Hongxing Wang^1^, Fuxiang Bai^3^, Lu Ding^1^, Yanyan Huang^2^, Changchang Lu^2^, Siyuan Chen^1^, Chunyang Li^4^, Xuetian Yue^5^, Xiaohong Liang^1^, Chunhong Ma^1^, Liyun Xu^2*^, Lifen Gao^1*^

1. MATERIALS AND METHODS **Cell culture and Reagents**

The human NSCLC cell lines A549 and NCI-H1975 were purchased from the Shanghai Cell Collection of Chinese Academy of Sciences (Shanghai, China). They were maintained in Dulbecco’s Modified Eagle’s Medium (DMEM; C11995500BT, GIBCO, New York, USA) supplemented with 10% fetal bovine serum (FBS; 10099141, GIBCO), 100 U/ml penicillin, 100 mg/ml streptomycin and 2 mM L-glutamine and incubated at 37°C in an incubator with 5% CO_2_ and saturated humidity. Human IL-6 was bought from PeproTech (200-06, Texas, USA). The antibody for TIM-4 and matrigel were purchased from Sigma-Aldrich (St. Louis, USA). D-Luciferin was purchased from BioVision (7903, Heinrichstr., Switzerland).

**Establishment of TIM-4-knockdown cells**

Stable cell lines with TIM-4 knockdown in A549 and H1975 cells were established after infecting with viral supernatants containing a recombinant Lentivirus Vector - LV-shTIM-4-GFP (LV-shTIM-4, Genechem, Shanghai, China) at 20 multiplicity of infection. After 8-12 h, cells were washed with DMEM to remove the complexes, and 1 ml fresh 10% FBS-containing DMEM were added. Cells were incubated in a CO_2_ incubator at 37°C for an additional 3 days and selected with 12 μg/ml puromycin for at least 1 week, then stable clones were maintained in 1 μg/ml puromycin, and harvested for experiments *in vitro*. The target sequence for shTIM-4 was 5’-AACCTATTGTTCGCAGAAA-3’.

**Immunohistochemistry (IHC) and Evaluation**

IHC was performed as reported in previous studies. Two serial 3- to 5-μm-thick tissue sections were prepared from each tumor specimen for TIM-4 and IL-6 staining. In the process of staining, sections with primary antibodies replaced by phosphate-buffered saline (PBS) served as the negative control. The results of IHC staining with anti-TIM-4 (Abcam, Cambridge, USA) and anti-IL-6 (Abcam) were evaluated by two independent experienced pathologists according to a semi quantitative grading system on the proportion of stained cells and their intensity. In this study, TIM-4 or IL-6 expression was defined in tumor cells. Staining intensity was scored as: 0 (negative), 1 (weak), 2 (moderate), or 3 (strong). The percentage of positive tumor cells was scored as: 0, for 5%; 1, for 5-25%; 2, for 26-50% 3, 51-100%. A histological score of the entire tissue section were generated as the product of intensity and the percentage of positive tumor cells. The results of IHC staining were divided into two groups. Tumors scored as 0 or 1 were considered “low group”, and those scored as 2 or 3 were classified as “high group”.

**Transwell migration assay**

Brieﬂy, 5~8×10^^4^ lentivirus-infected A549 and H1975 cells in 200 μl serum-free medium were added to the upper chambers of transwell plate and treated with IL-6 or not, and the lower chambers were filled with 600 μl medium containing 10% FBS as chemoattractant. 24 h later, the non-migratory cells on the upper surface of the chambers were removed by a cotton swab, and the migrated cells were fixed in 4% paraformaldehyde and stained with 1% crystal violet. Then, at least five randomly fields were selected and observed by microscope.

**Matrigel invasion assay**

Matrigel was diluted 1:4 with DMEM. Transwell filters were coated with 30 μl diluted matrigel for 30 minutes at 37°C in an incubator. 6~10×10^^4^ lentivirus-infected A549 and H1975 cells in 200 μl serum-free medium were added to the upper chamber with or without IL-6 treatment. The lower chamber was filled with 600 μl medium containing 10% FBS. After incubation for 24 h, cells were treated as same as the procedures for the migration assay.

**Quantitative real-time PCR (qPCR) analysis**

Total RNA from cells treated as indicated was extracted by phenol-chloroform and precipitated by ethanol, and all RNA was DNase treated before reverse transcription. 2-3 μg of total RNA was used for cDNA synthesis with RevertAid First Strand cDNA Synthesis Kit (Thermo Fisher, Waltham, USA). qPCR was performed with SYBR Green Real time PCR Master Mix (Applied Biosystems, Waltham, USA), and was run on ABI 7500 Real-time PCR system.

| Primers | F (5’-3’)  R (5’-3’) |
| --- | --- |
| hN-cadherin | CAGACATGGAAGGCAATCCCACA  CTGGATGGCGAACCGTCCAGTAGGA |
| hE-cadherin | ACAGCCCCGCCTTATGATT  TCGGAACCGCTTCCTTCA |
| hVimentin | GCTGAATGACCGCTTCGCCAACT  GCTCCCGCATCTCCTCCTCGTA |
| hSlug | TTCGGACCCACACATTACCT  GCAGTGAGGGCAAGAAAAAG |
| hTIM-4 | ACAGGACAGATGGATGGATGGAATACCC  AGCCTTGTGTTTCTGCG |
| hActin | AGTTGCGTTACACCCTTTC  CCTTCACCGTTCCAGTTT |

**Western Blot (WB) analysis**

Cells were washed once with PBS (phosphate buffered saline), and lysed in RIPA lysis buffer (Beyotime, Shanghai, China) with 1% protease inhibitor cocktail, and 1% phosphatase inhibitor (Sigma-Aldrich). Proteins (30 μg) were separated with 12% SDS-PAGE, and transferred 0.45 μm PVDF membranes were blocked by BAS and probed with appropriate antibodies.

**Antibodies**

Antibodies used in this article were listed as follows: Rabbit anti-TIM-4 (Sigma, SAB3500444 and HPA015625, 1:1000 for WB); Rabbit anti-Stat3 (Cell Signaling Technology, #8232, 1:1000 for WB, Massachusetts, USA); Rabbit anti-Phospho-Stat3 (Cell Signaling Technology, #9145, 1:1000 for WB); Rabbit anti-NF-κB p65 (Cell Signaling Technology, #8242, 1:1000 for WB); Rabbit anti-Phospho-NF-κB p65 (Cell Signaling Technology, #3033, 1:1000 for WB); Rabbit anti-Slug (Cell Signaling Technology, #9585, 1:1000 for WB); Rabbit anti-N-cadherin (Proteintech, 22018-1-AP, 1:2000 for WB, Chicago, USA); Rabbit anti-E-cadherin (Bioworld Technology, BS90443, 1:1000 for WB, Louis Park, USA); Mouse-anti-beta Actin (Abcam, ab8226, 1:2000 for WB).

**Flow cytometry**

A549 and H1975 cells were collected and washed twice with PBS, then they were adjusted to a cell count of 1×10^^6^/tube. The cells were labeled with the corresponding antibody by incubation at 4 °C for 30 minutes, then washed once with PBS, and finally fixed with 1% paraformaldehyde. The CytoFLEX flow cytometer (BECKMAN, Indianapolis, USA) was used for detection and analysis. PE-conjugated anti-human TIM-4 (354004, BioLegend).

**ELISA**

Supernatants from cultured cells were collected and assayed to detect IL-6 production by ELISA (1110602, Dakewe, Shenzhen, China). All assays were performed in triplicate and were repeated 3 times under independent conditions.

**Luciferase reporter assay**

The promoter region of TIM-4 corresponding to -1247~+300 was cloned from PBMCs of healthy controls by PCR using forward 5’-CGGGGTACCTGCTTTCCTCAAATGTCTGATG-3’ and reverse 5’-GGAAGATCTATAAACCTCTGTGTAACAAGGC-3’ primers. The PCR fragment was then cut with Bgl II and Kpnl restriction endonucleases, and cloned into Bgl II/Kpnl treated pGL3-Basic-Luciferase vector. The luciferase assay was performed in transfected A549 and H1975 cells with pGL3-hTIM-4 or pGL3-Basic plasmids accompanying by pRL-TK, respectively. After 36 h, cells were treated by IL-6 or NF-κB inhibitor for another 12 h. 48 h later, cells were harvested with passive lysis buffer. Supernatants were used to detect both firefly and renilla luciferase activity by using Dual-Glo Luciferase assay system (Promega, Madison, USA) on a microplate reader (TECAN, Männedorf, Switzerland). The ratios of firefly luciferase/renilla luciferase measurements were used for correction.

**2. SUPPLEMENTAL FIGURES**

**
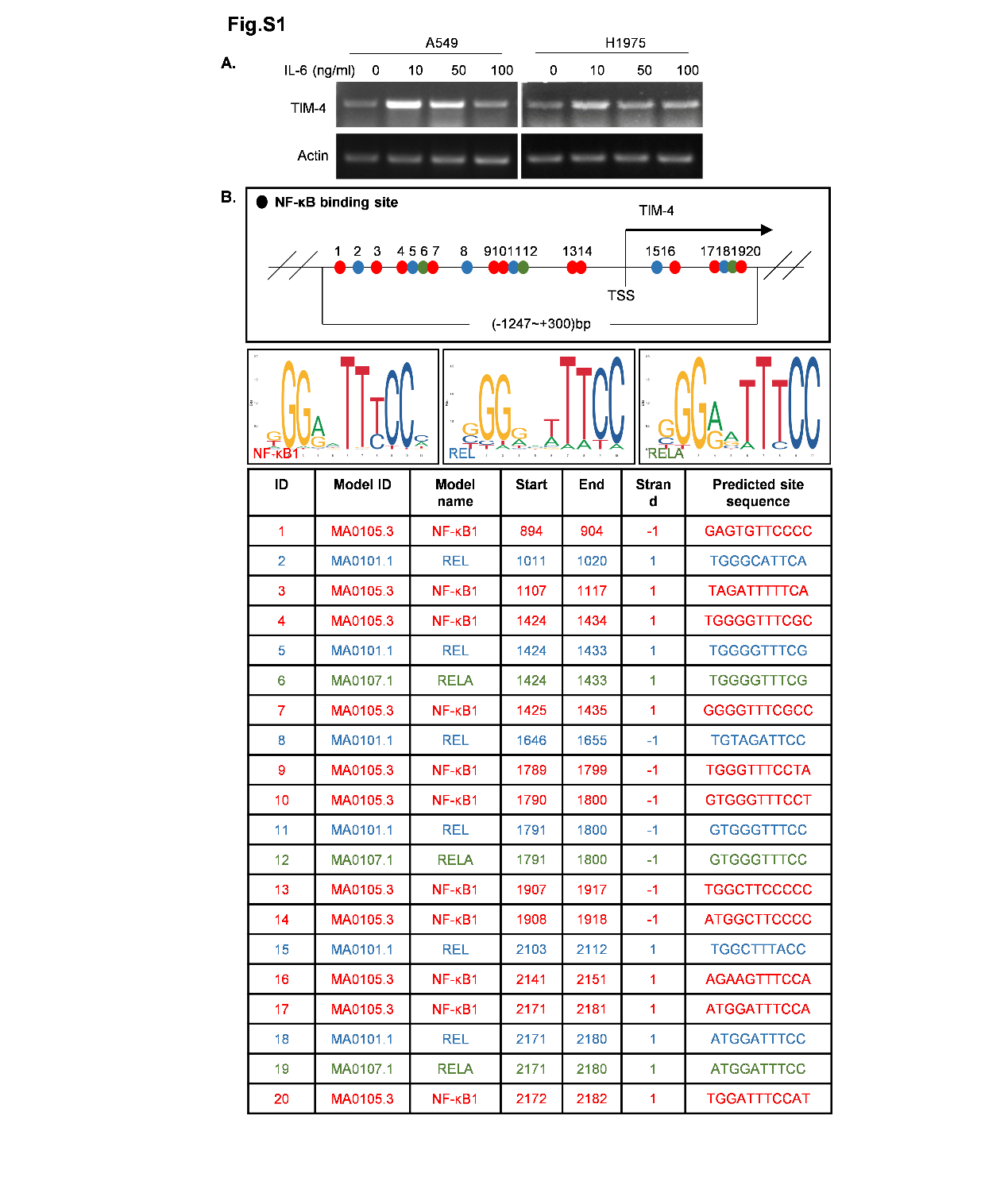
**

**Figure S1** IL-6 promoted TIM-4 expression in a dose-dependent manner. (**A**) TIM-4 mRNA were detected by RT-PCR with IL-6 stimulation in different concentration (0, 10, 50, 100 ng/ml) in both A549 and H1975 cells. (**B**) Pattern diagram of NF-κB binding sites predicted by PROMO and JASPAR software to the TIM-4 promoter (−1247~+300 bp).


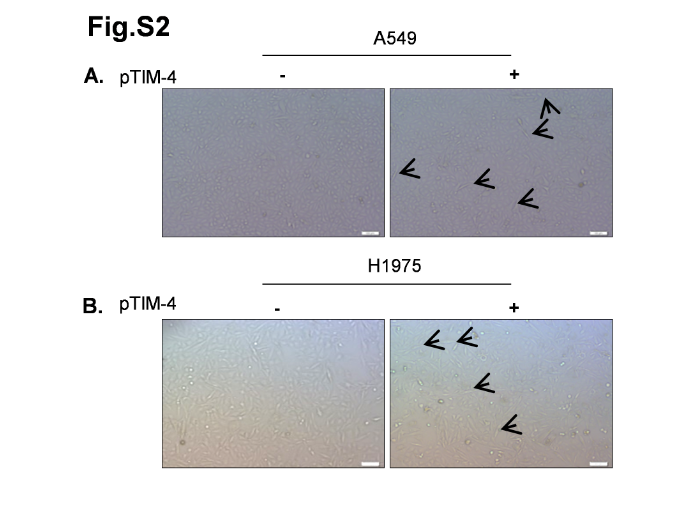


**Figure S2** Changes in cell morphology. Changes in A549 (**A**) and H1975 cells (**B**) morphology after transfected with pTIM-4 and pcDNA3, respectively. Bar, 100 μm.


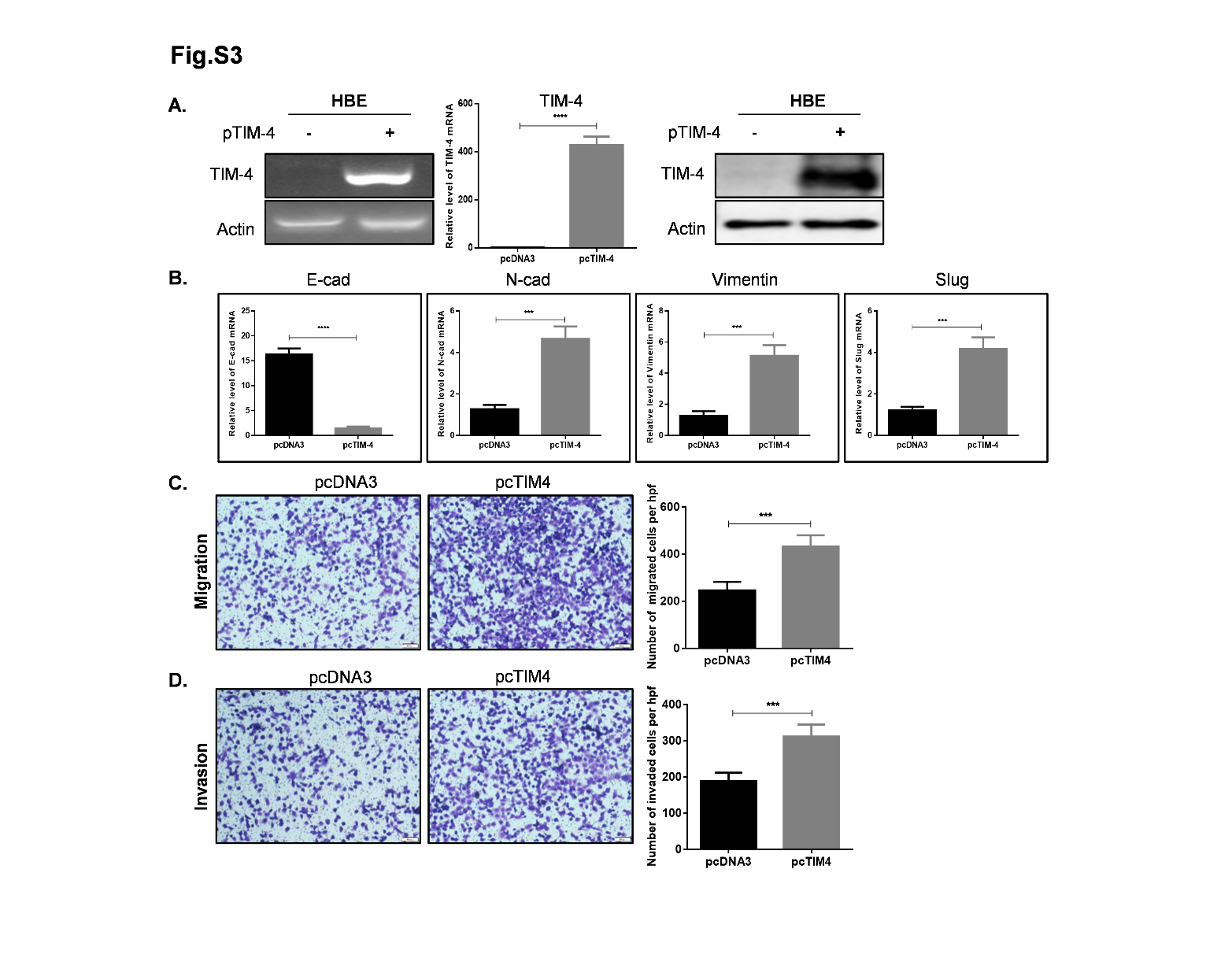


**Figure S3** TIM-4 overexpression promoted EMT, migration and invasion of non-cancer human bronchial epithelial cell line HBE. (**A**) Overexpressed efficiency of TIM-4 was verificated by RT-PCR, qPCR and western blot. (**B**) EMT related genes E-Cad, N-Cad, Vimentin and Slug were assayed in TIM-4 overexpressed HBE cells by qPCR. Migration (**C**) and invasion (**D**) abilities were detected in TIM-4 overexpressed HBE cells by transwell assay. The migrated and invasive cells were photographed (Bar, 100 μm). Representative pictures were shown. Data in **A**, **B**, **C**, and **D** were shown as median ± SD of three independent experiments. ****P* <0.001, *****P* <0.0001, by 2-tailed Student’s *t* test.


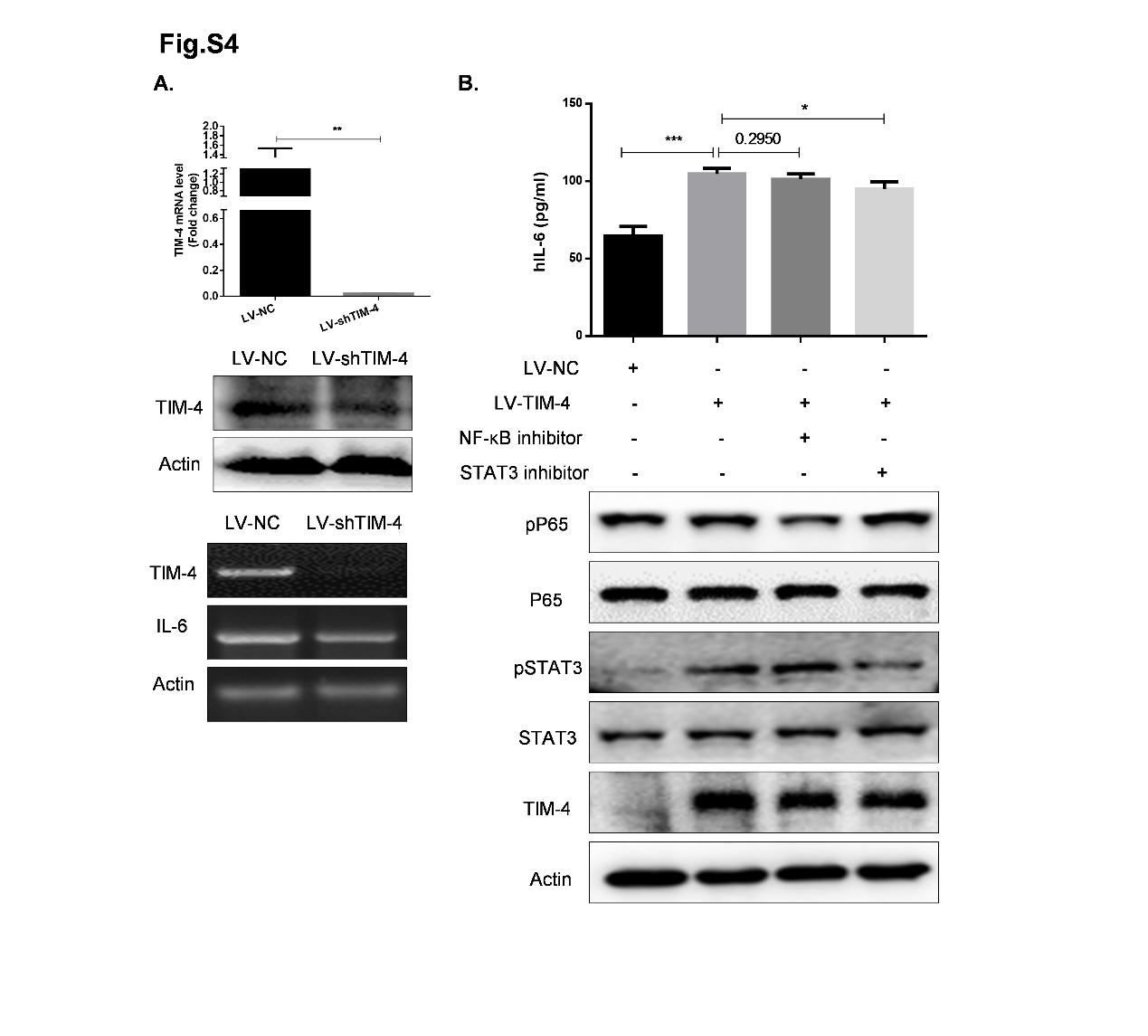


**Figure S4** TIM-4 overexpression increased IL-6 production via STAT3. (**A**) Interference efficiency of TIM-4 was verificated by qPCR and western blot (upper), The transcriptional level of TIM-4 and IL-6 was detected in A549 with TIM-4 knockdown stably by RT-PCR (bottom). (**B**) NF-κB or STAT3 inhibitor was used to stimulate LV-NC and LV-TIM-4-A549 cells, and pP65, pSTAT3 and TIM-4 protein expression were detected by western blot, and the secretion of IL-6 in supernatants was detected by ELISA. Data in **A** and **B** are shown as median ± SD of three independent experiments. **P*<0.05, ***P*<0.01, ****P* <0.001, by 2-tailed Student’s *t* test.


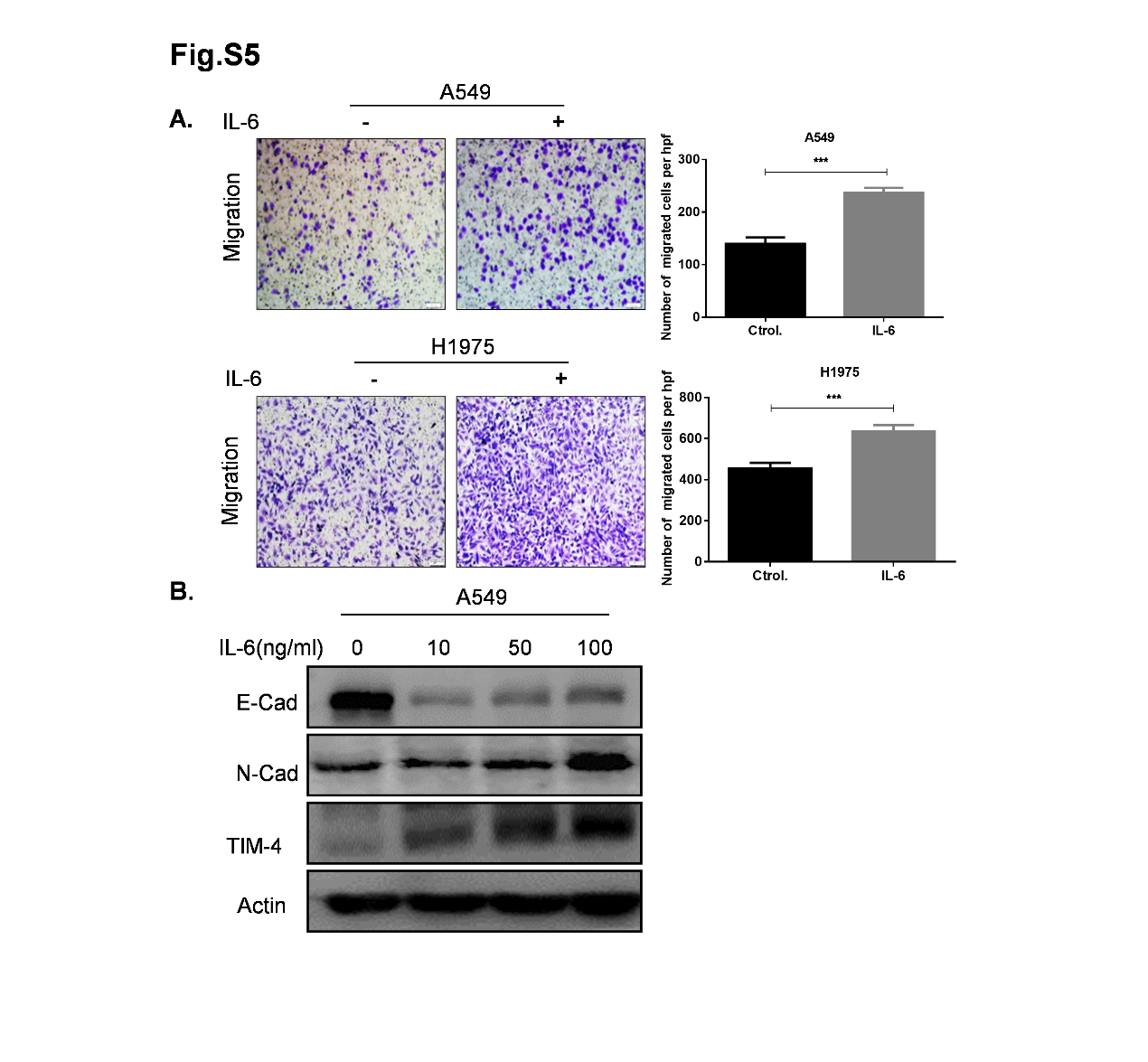


**Figure S5** IL-6 promoted migration and EMT of NSCLC cells. (**A**) IL-6 was used to stimulate A549 and H1975 cells, and transwell assay was performed to detect migration ability. (**B**) E-Cad, N-Cad and TIM-4 were detected by western blot. Bar, 100 μm. Data in **A** is shown as median ± SD of three independent experiments. ****P* <0.001, by 2-tailed Student’s *t* test.


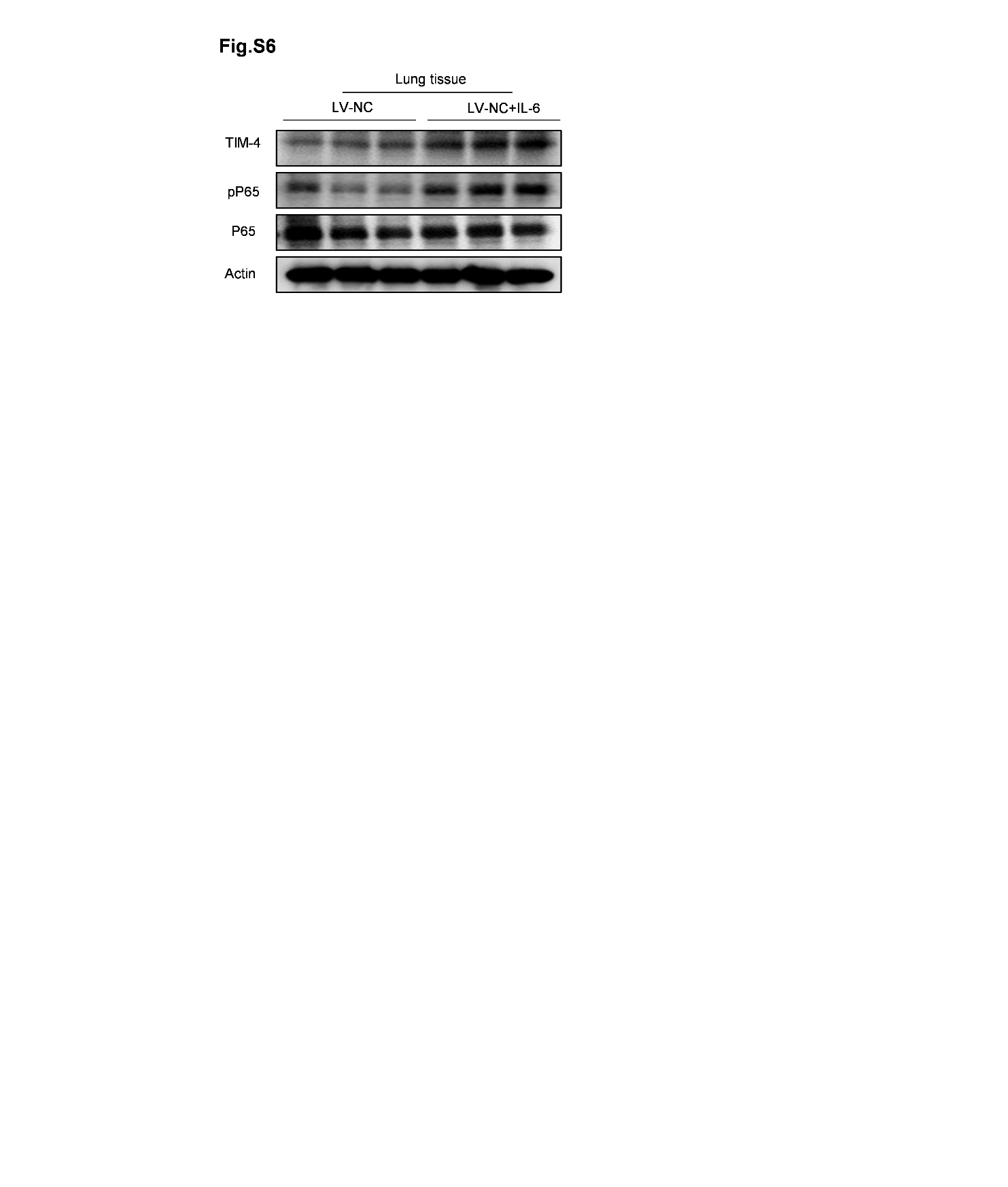


**Figure S6** IL-6 promoted the expression of TIM-4 and pP65 in lung cancer metastasis tissues. The expression of TIM-4 and pP65 were detected in lung cancer metastasis tissues from LV-NC and LV-NC+IL-6 groups by western blot.
